# Patient engagement with antibiotic messaging in secondary care: a qualitative feasibility study of the ‘review and revise’ experience

**DOI:** 10.1186/s40814-020-00590-5

**Published:** 2020-04-04

**Authors:** Fiona Mowbray, Katy Sivyer, Marta Santillo, Nicola Jones, Tim E. A. Peto, A. Sarah Walker, Martin J. Llewelyn, Lucy Yardley

**Affiliations:** 1 Centre for Clinical and Community Applications of Health Psychology, University of Southampton, Southampton, UK; 2 Nuffield Department of Medicine, University of Oxford, Oxford, UK; 3 Oxford University Hospitals NHS Foundation Trust, Oxford, UK; 4 Brighton and Sussex Medical School, Falmer, UK

**Keywords:** Antibiotic prescribing, Hospital patients, Antimicrobial stewardship

## Abstract

**Background:**

We aimed to investigate and optimise the acceptability and usefulness of a patient leaflet about antibiotic prescribing decisions made during hospitalisation, and to explore individual patient experiences and preferences regarding the process of antibiotic prescription ‘review and revise’ which is a key strategy to minimise antibiotic overuse in hospitals.

**Methods:**

In this qualitative study, run within the feasibility study of a large, cluster-randomised stepped wedge trial of 36 hospital organisations, a series of semi-structured, think-aloud telephone interviews were conducted and data were analysed using thematic analysis. Fifteen adult patients who had experienced a recent acute medical hospital admission during which they had been prescribed antimicrobials and offered a patient leaflet about antibiotic prescribing were recruited to the study.

**Results:**

Participants reacted positively to the leaflet, reporting that it was both an accessible and important source of information which struck the appropriate balance between informing and reassuring. Participants all valued open communication with clinicians, and were keen to be involved in antibiotic prescribing decisions, with individuals reporting positive experiences regarding antibiotic prescription changes or stopping. Many participants had prior experience or knowledge of antibiotics and resistance, and generally welcomed efforts to reduce antibiotic usage. Overall, there was a feeling that healthcare professionals (HCPs) are trusted experts providing the most appropriate treatment for individual patient conditions.

**Conclusions:**

This study offers novel insights into how patients within secondary care are likely to respond to messages advocating a reduction in the use of antibiotics through the ‘review and revise’ approach. Due to the level of trust that patients place in their care provider, encouraging HCPs within secondary care to engage patients with greater communication and information provision could provide great advantages in the drive to reduce antibiotic use. It may also be beneficial for HCPs to view patient experiences as cumulative events that have the potential to impact future behaviour around antibiotic use. Finally, pre-testing messages about antibiotic prescribing and resistance is vital to dispelling any misconceptions either around effectiveness of treatment for patients, or perceptions of how messages may be received.

**Trial registration:**

Current Controlled Trials ISRCTN12674243 (10 April 2017),

## Background

Antimicrobial resistance (AMR) is an important issue patients worldwide, with impacts on both healthcare costs and patient safety [1]. Over prescribing of antimicrobials contributes significantly to the growing problem of AMR worldwide [2]. Up to 50% of antibiotic prescribing may be inappropriate either because antibiotics are not indicated or the agent(s) selected are too broad or continued longer than needed [3–5]. In primary care, efforts to minimise antibiotic overuse are directed at only starting antibiotic treatment when there is a clear clinical reason to do so [6]. In secondary care, where patients are more acutely unwell, strategies to optimise antibiotic use involve prompt empiric antibiotic therapy while there is diagnostic uncertainty, followed by regular review and revise to target and where appropriate, stop antibiotic treatment. In the NHS (National Health Service), this strategy is set out in Department of Health guidance, ‘Start Smart then Focus’ [7]. Start Smart then Focus recommends five decisions prescribers can take reviewing antibiotic therapy: stop, continue, move IV to oral, broaden or de-escalate or move to outpatient intravenous therapy. However, controlling antibiotic overuse through review and revise is challenging [8–10].

Antibiotic Review Kit (ARK) Hospital is a complex behavioural intervention targeting all healthcare professionals (HCPs) involved in prescribing, dispensing or administering antibiotics for acute and general medicine adult patients. This paper reports the findings of a set of interviews with patients as part of the wider developmental and feasibility work for a full-scale RCT (randomised controlled trial) aiming to encourage appropriate and timely stopping of antibiotics that are no longer needed. The overall intervention incorporates digital, behavioural and organisational elements, including online training, a decision aid tool to support decision-making around antibiotic prescriptions, a patient information leaflet, a structure for monitoring and discussing implementation of the intervention, detailed implementation guidance, a resources website and a peer support network [11]. For the feasibility trial, all intervention elements were implemented in one medium-sized acute hospital in the UK. Full details of how ARK was used by healthcare professionals during the study are available in a separate publication [12]. The qualitative study described here was an investigation of the feasibility and acceptability of the patient leaflet element of the intervention among patients at the feasibility study site. This paper details the development and optimisation of the leaflet. The full protocol for the main trial is reported elsewhere [13].

Evidence from primary care suggests that engaging patients in antibiotic-prescribing decisions can facilitate reducing antibiotic use [14]. In secondary care, while there is evidence that both patients and clinicians want an increase in shared decision-making around prescribing [15, 16], it is not yet clear whether this shared decision-making could lead to similar reductions in antibiotic use [17]. As a result, the ARK-Hospital information leaflet aimed to reassure, inform and empower patients about potential changes made to their antibiotic prescription. However, there is an absence of research evidence to inform the design and use of a patient information leaflet to support the antibiotic ‘review and revise’ prescribing process within secondary care.

The aim of this qualitative study was to investigate and optimise the acceptability and usefulness of such a patient leaflet in secondary care, ahead of intended use in a full-scale RCT. We also aimed to explore and understand individual patient experiences of the ‘review and revise’ process and identify patient views and preferences regarding antimicrobial treatment in hospitals to inform both the larger trial and any future research in this field.

## Methods

### Developing the patient information leaflet

The detail and planning of the ARK-Hospital intervention are described elsewhere [11]. The patient leaflet was developed iteratively, building initially on previous research (GRACE-INTRO) which drew on theory and qualitative user feedback as detailed elsewhere [18] and was designed to be understood by readers with lower levels of health literacy. This was further refined by health psychologists and clinicians to ensure accuracy of the health messages. Feedback was sought from project stakeholders and from members of a public and patient involvement (PPI) group. This feedback included suggestions for ways to improve the look and feel of the leaflet, e.g. by incorporating more engaging images, simplifying the layout and making minor clarifications to the text. PPI input was particularly useful in ensuring that the leaflet gave relevant, but accessible information about antibiotic resistance and how to present this without causing undue concern. The leaflet provides patients with brief information about when antibiotics are used, the possible risks of taking antibiotics, the ‘review and revise’ process and advice about what to do when their antibiotics are stopped.

### Recruitment

Ethical approval for the ARK-Hospital implementation study (ISRCTN: 12674243) was obtained from the National Research Ethics Committee (REC reference 17/SC/0034), including feasibility, pilot and main trial phases. It is useful to note that for the feasibility, pilot and main trials, neither staff, nor patients are individually consented into the study as the overall unit of randomisation and analysis is the site or trust and no data is identifiable. Only for qualitative date collection did we consent staff or patients. As such, participants for this qualitative component were recruited as a convenience sample from patients admitted through the Acute Medical Unit (AMU) at the feasibility study site (the Royal Sussex County Hospital, Brighton) between June 2017 and February 2018. All participants had been prescribed antibiotics during their hospital stay. For most patients, the intervention leaflet was given to patients at their time of discharge from hospital, though in a few cases patients received the leaflet when a change had been made to their antibiotic prescription. In line with ethics requirements, participants were identified and invited to take part in the study at the time of discharge by medical staff who introduced the study and provided them with a study information sheet explaining that participation was both confidential and voluntary. Medical staff also checked that the participant had been given a copy of the leaflet and asked them to keep this for the interview. Interested participants completed the consent form and provided contact details to the member of medical staff who then posted these details to researchers at the University of Southampton. Researchers then contacted participants to arrange an interview and verbal consent and demographic data were collected prior to each interview. A total of 125 patients were approached about the study, with 25 providing consent to be contacted by a researcher. Of these 25 patients, 10 dropped out, either because they no longer wanted to take part by the time of interview, or because they could not be contacted. This left a total of 15 study participants.

### Interviews

**Fig. 1:**
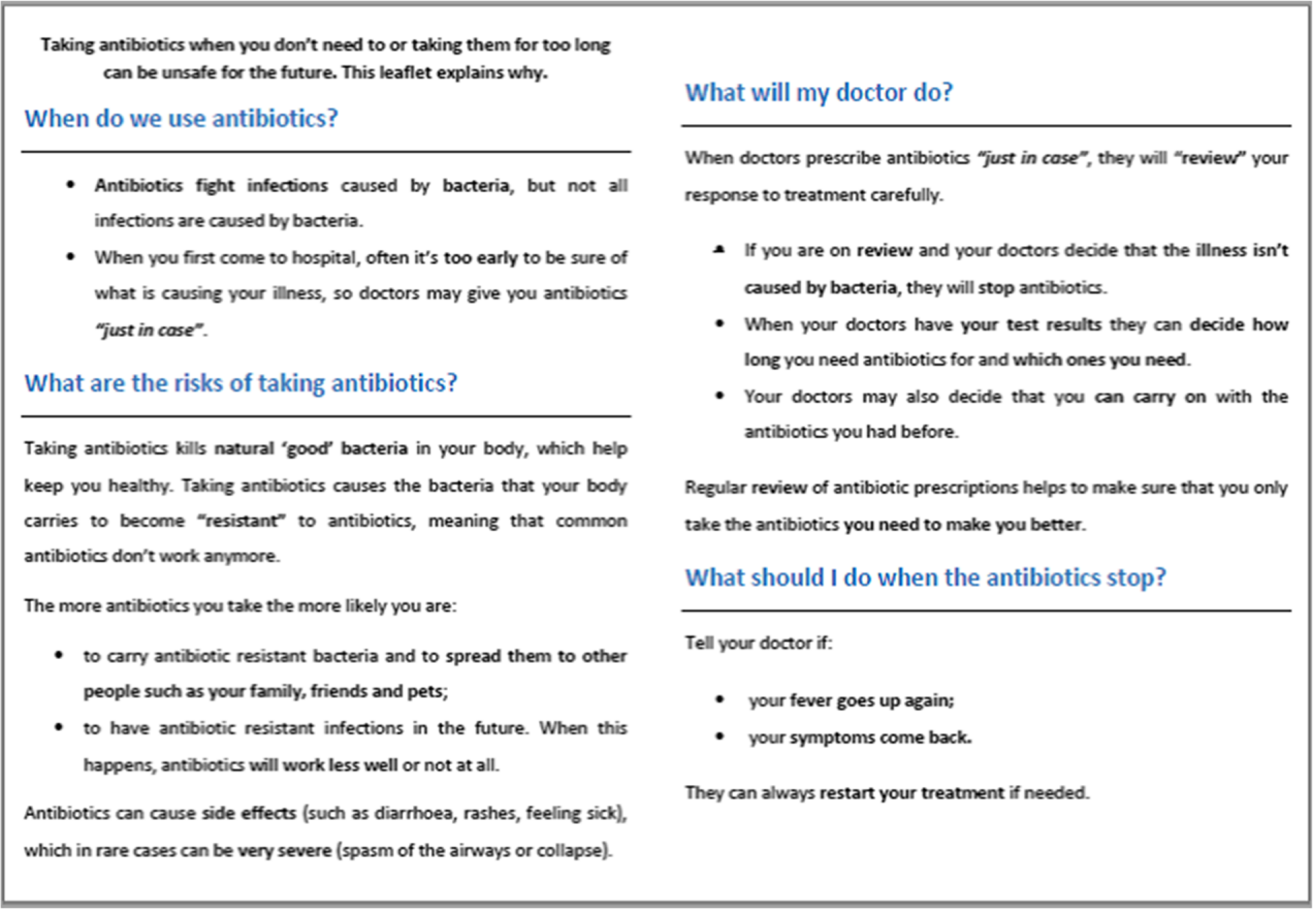
Original version of leaflet text

**Fig. 2:**
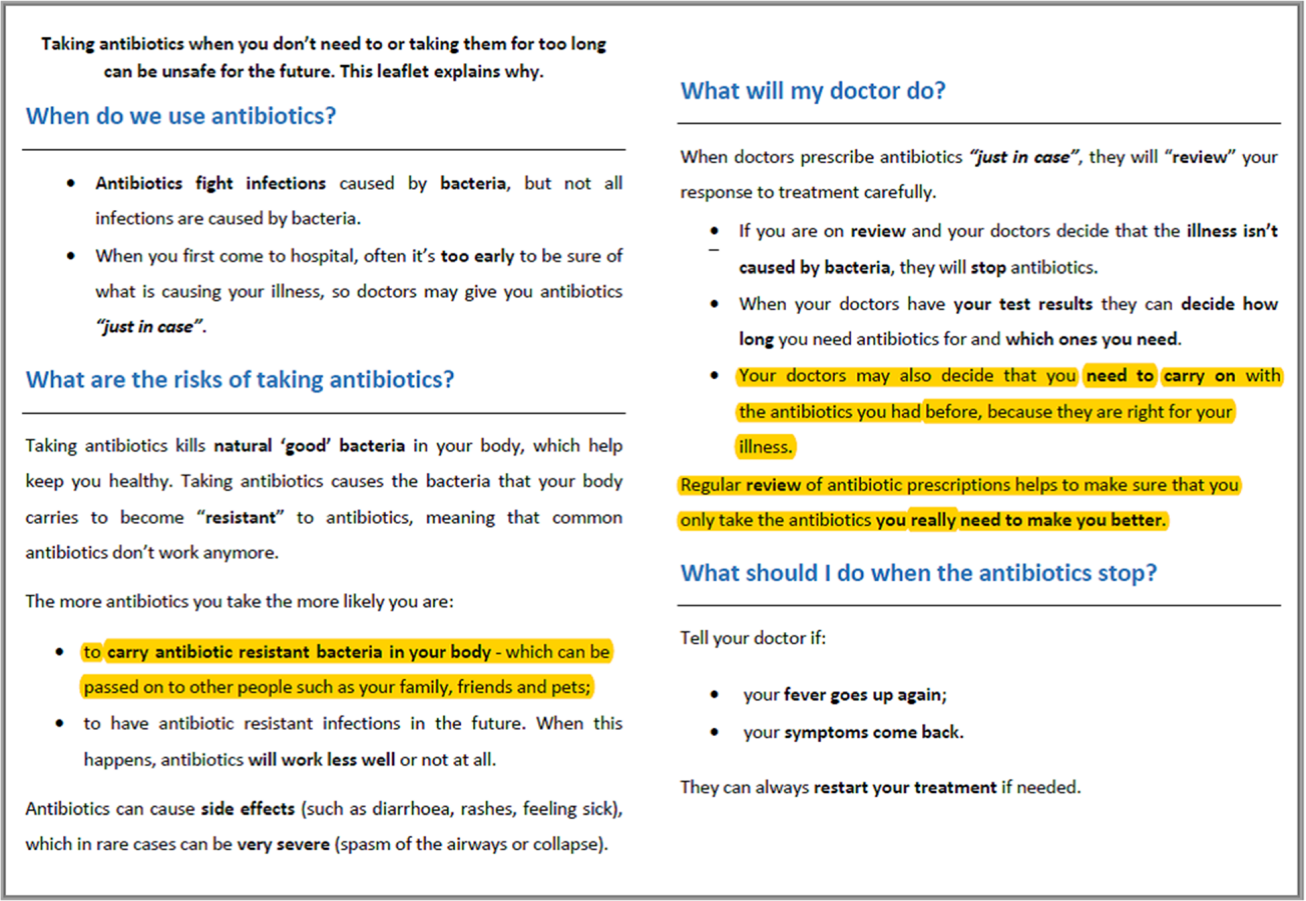
Final version of leaflet text following revisions. Highlighting indicates areas where text was altered as a result of qualitative data and PPI input

The study methodology involved semi-structured, think-aloud [19], telephone interviews, which lasted between 20 and 30 min. These were conducted by FM and KS, who are PhD qualified, research fellows with training and experience of qualitative methods in health research, including conducting cognitive interviews. The study participants were not acquainted with the researchers prior to the study, but they were informed about the purpose of the study and were made aware that the researchers were affiliated with University of Southampton. Participants were initially asked a series of open questions to explore their experience and perception of the ‘review and revise’ process, including any changes that were made to their antibiotic prescription and perceptions about the duration of antibiotic treatment. They were then asked to read, or listen to the interviewer read, the patient leaflet (Fig. 1) that they had received while in hospital or at the time of discharge. Participants were asked to say everything that they were thinking out loud while they read the leaflet. Several more open-ended questions followed, which explored what participants liked or disliked about the leaflet, what they viewed as most relevant, and any suggested changes to improve the leaflet. Using a think aloud methodology enabled us to explore participant reactions to the leaflet and gain detailed feedback about each aspect of the intervention, allowing us to make changes to and optimise the content. As negative feedback is especially helpful in developing the most effective messages, we deliberately elicited this within our study. After an initial nine interviews, the leaflet was revised (Fig. 2) based on participants’ feedback before being tested with a further 6 participants, for a total of 15 unique participant interviews. Participants were compensated with a £10 shopping voucher for taking part in the study.

### Data analysis

Interviews were audio recorded and transcribed verbatim. No field notes were made by researchers either during or after the interviews and transcripts were not returned to participants. Analysis initially focused on identifying any potential barriers to use of the leaflet and interview feedback was used to identify any areas where changes might make it more acceptable, engaging or useful. Each transcript was reviewed line-by-line to draw out all responses that were either positive or negative perceptions of the leaflet [20]. Responses were tabulated and each negative comment was reviewed to determine whether a change was necessary. If so, the solution was recorded in the table, discussed with the wider team and the change was made. Changes were made if they were likely to impact on the acceptability of the leaflet or the ‘review and revise’ process. This included exploring aspects such as whether the information was perceived as convincing, reassuring and comprehensible. The MoSCoW (must have, should have, could have, would have) criteria were used to assess priority [20] and each change was made in line with the common and intervention specific guiding principles of the person-based approach [21]. Although similar to content analysis, the table of changes as illustrated below, has been created specifically for use in intervention development. As such, it does not aim to quantify qualitative data, but instead offers a way to analyse this intervention feedback in a systematic and efficient manner, often running in parallel with in depth thematic analysis [21]. An example of the data tabulation is shown in Table 1.

**Table 1:** Example of table of iterative changes made to patient leaflet

Page or aspect of the intervention	Positive comments	Negative comments	Possible change	Reason for change	Agreed change	MoSCoW
Section titled: ‘What are the risks of taking antibiotics?’		Confusion over how antibiotic resistant bacteria can be spread to others, e.g. ‘I didn’t realise that antibiotic resistance can spread to other members of the family. I’m not quite sure what it means.’	**Explanation of spreading antibiotic resistant bacteria to others made clearer.**	**Important to behaviour change** as we do not want to confuse or concern patients. **Expert** clinicians and health psychologists agreed the change was suitable. **Repeatedly** mentioned by participants.	Changed bolded text to reduce any concerns and clarified text about passing on resistance to others.	**Must have**, crucial to ensure patients accurately understand the risks of antibiotics.

Each transcript also underwent inductive thematic analysis [22], supported by use of the QSR NVivo 11 software, and was coded into emerging themes, which represented frequent patterns of meaning within the dataset. Coding followed the aims of the research, focusing on patients’ experiences and perceptions of the ‘review and revise’ process and the acceptability of a patient leaflet. Coding was done by FM, an experienced qualitative researcher, with KS reviewing transcripts and codes frequently and advising on the development of themes. The final themes were agreed upon by the research team through discussion and consensus that saturation had been reached based on the completion of 15 interviews. Data collection stopped when no new concerns or themes emerged.

## Results

Ten (67%) women and five (33%) men participated, with an age range of 50–91 and mean age of 72 (SD = 13.2). All participants spoke English as their first language and reported their cultural background as British. Participants had all been discharged from hospital and eight (53%) were still taking antibiotics at the time of discharge. Full demographic details are available in Table 2.

**Table 2:** Demographic characteristics of the sample (*n* = 15)

Demographic characteristics	Number/proportion of the sample *n* (%)
**Gender**	
Female	10 (67%)
Male	5 (33%)
**Age**	
18–34 years	0 (0%)
35–54 years	3 (20%)
55–74 years	3 (20%)
> 75 years	9 (60%)
**Cultural background**	
White British/English	15 (100%)
Other	0 (0%)
**Education**	
GCSEs/GNVQs or equivalent	6 (40%)
A-levels	2 (13%)
University degree (e.g. BSc, BA, MSc, PhD)	1 (7%)
No exams taken	6 (40%)
Other	0 (0%)
**Languages spoken**	
English	15 (100%)
Other	0 (0%)
**Taking antibiotics when discharged**	
Yes	8 (53%)
No	5 (33%)
Cannot remember/not sure	2 (13%)

Following detailed thematic analysis, 34 subcategories that fell into 12 categories were extracted from the transcripts. From these, 4 interlinking themes were identified (Table 3). The participants described their perceptions of the leaflet and the impact that it had on their views of treatment. This led to discussions about their largely positive experiences of the ‘review and revise’ process, while also linking to any existing knowledge of antibiotics and antibiotic resistance. Finally, participants all described the trust that they place in HCPs to make treatment decisions, which appeared to mitigate any potential concerns around prescriptions being changed or stopped.

**Table 3:** Analytical framework for developing categories and themes for patients’ experiences

Category	Definition	Example quote
**Theme 1: leaflet acceptability and impact on perceptions of treatment**		
*Positive perceptions of review and revise*	Positive feedback given about the leaflet as an introduction to the ‘review and revise’ process. Also includes discussion about recommending the leaflet to others and the overall relevance of the leaflet.	‘I think it makes you feel better knowing that you’re being checked on and deciding whether we’re going to need all these antibiotics all the time.’ (P9, female, 81)
*New concerns raised about resistance*	Any concerns or questions that patients discussed regarding antibiotic resistance as well as how this may impact friends and family.	‘The one thing that would probably worry me more than anything is that the more antibiotics you take the more likely you are to spread them to other people, such as your family and friends.’ (P3, male, 78)
*Timing of when leaflet received*	Discussion about perceptions of the impact that timing of the leaflet had on their input into treatment as well as perceived relevance of the leaflet.	‘I found that where I had the leaflet it was very helpful in actually talking to them [HCPs] about what I was being specifically treated for.’ (P3, male, 78)
**Theme 2: experience of review and revise process**		
*Positive perceptions of initial antibiotic prescribing*	Positive perceptions about how antibiotics were initially prescribed, including reasons for hospitalisation, drug mode of delivery, awareness (or not) of initial prescription and any information given about prescription and/or treatment.	‘…when they put me on the antibiotics they were telling me exactly what they for, how long I was going to be on for, and what they was doing, and if I’ve got any problems with them at all let them know and they’d stop them.’ (P5, male, 77)
*Experience of prescription changes*	Feedback about any changes to antibiotic prescription. Includes discussion about any diagnostic testing and results, changes to drug mode of delivery and the efficacy of treatment.	‘They started me on antibiotics and I had about 2 or 3 that day and then 2 in the morning, and then when they gave me an x-ray they realised it wasn’t a chest infection, they think it was a viral infection. So they cancelled the antibiotics.’ (P4, female, 51)
*Patient perceptions of input into treatment*	Amount of input patients felt they had regarding antibiotic treatment. Reflections on whether they had the opportunity to ask questions or discuss treatment at the time of prescribing, or as any changes to treatment were made, up until the time of discharge.	‘Anything I did want to know, people automatically told me if I had anything [medications], which was really good.’ (P14, female, 83)
**Theme 3: existing knowledge of antibiotics**		
*Positive past experience*(*s*) *of antibiotic treatment*	Any positive past treatment experiences reported by patients. It includes aspects of how treatment was received, but also treatment efficacy.	‘Well obviously, the only thing I use them for is if you’ve got an infection because then it kills the infection; it makes you well again. That’s the only thing I know about antibiotics.’ (P4, female, 51)
*Negative past experience*(*s*) *of antibiotic treatment*	Any negative past experiences of antibiotic treatment, with discussion including problems with treatment, particularly the experience of side effects.	‘I agree that some antibiotics aren’t great, and I know in the past I’ve had antibiotics that upset your stomach and had to stop them or change them. So I’ve said in the past, don’t give me that one because I don’t like it.’ (P1, female, 50)
*Existing concerns about antibiotic resistance*	Patients’ existing knowledge of antibiotic resistance and the concerns that they had about this.	‘You can get immune to them if you take too many. I mean it’s pretty obvious, it’s like anything else, that they will stop working, that’s why I don’t like to take so many.’ (P2, female, 65)
**Theme 4: trust in healthcare professionals**		
*Positive existing relationship with HCPs*	Positive perceptions that patients have about their relationship with HCPs, including previous experience of care by GPs and pharmacists, as well as positive experiences of care during their recent hospital stay.	‘The doctors and the hospital have been very good, because I have been admitted quite a few times. They don’t turn around and say oh no, not you again, they do treat me as a new patient every time.’ (P2, female, 65)
*Willingness to take antibiotics*	Specific discussions about being happy to take antibiotic medications in hospital, particularly as this is often life-saving and not always viewed as a ‘choice’ if patients want to recover.	‘I understand the risk you have to take, but if you’re in a situation like I was, where it was life and death, you’re going to take a chance of taking antibiotics, because if I hadn’t taken them I would have died.’ (P7, male, 62)
*Positive perceptions of HCPs as experts*	Perception of HCPs as experts giving each patient the best possible treatment. Patients discussed being happy to follow expert HCP advice about antibiotic treatment, including treatment duration, changes to treatment, and not always needing to feel involved in initial antibiotic treatment decisions.	‘I’d be quite happy to accept whatever a doctor prescribed for me, because they’re the experts and I am not.’ (P6, female, 91)

This data is an extract of quotes derived from thematic analysis of interviews exploring participants’ experiences of the ‘review and revise’ process and provision of an information leaflet in secondary care.

### Leaflet acceptability and impact on perceptions of treatment

During initial interviews, several participants had questions or concerns regarding antibiotic resistance, particularly how this can be spread to others. For most, this stemmed from a lack of awareness that resistance can be passed on and a lack of clarity about how this happens:

I didn’t realise that resistance could spread to others…I’m not quite sure what that means, how can it spread…I don’t understand that. (patient 2, female, 65)

We felt that it was important to address these concerns by making minor revisions to the leaflet in consultation with the PPI group. These revisions aimed to reassure readers that when their doctor prescribes antibiotics only when really needed, this helps to reduce the likelihood of developing (and hence passing on) resistance. Following these revisions, further patient interviews indicated that although there was still a lack of awareness around the spread of resistance, concern appeared to have been mitigated:

I didn’t realise that antibiotic resistance, you know by me taking it, it could affect somebody else…it doesn’t concern me, I just didn’t realise that, but it’s very easy to understand. (patient 13, female, 74)

Overall, the majority of participants reacted positively to the leaflet, explaining that they found it ‘informative’ and ‘easy to read’. Several participants also discussed the importance of being given the information that was included in the leaflet:

I think it’s a good move to actually inform the public, not just patients, but the general public. To inform them about the dangers in the future of antibiotics not working. (patient 11, male, 77)

The leaflet was given to some participants when antibiotics were initially prescribed, and to others only at the time of discharge from hospital. A couple of participants who received the leaflet during discharge mentioned that they may have found it more useful at the time of treatment, but the majority felt that it was still of interest and relevance at the time of discharge. In fact, all participants reported that they would recommend the leaflet to others and several explained that they had kept it to show to family and friends, or as a document that they could refer back to for further information.

### Positive experience of ‘review and revise’ process

Participants all discussed details of their recent stay in the hospital, and reflected on their experience of the antibiotic ‘review and revise’ process. Many participants had been admitted for very serious conditions and spoke about being unaware of their initial antibiotic prescription. Others explained that they were started on antibiotics while diagnostic tests were conducted to confirm their diagnosis. Regardless of awareness of treatment or a confirmed diagnosis, all participants reported positive perceptions of the antibiotic prescribing process, often recognising the importance of receiving fast, initial treatment:

I was just told it was a precaution because it was suspected meningitis and obviously I think in that case they did the right thing, because meningitis is pretty nasty and can kill. (patient 1, female, 50)

Several participants had experienced changes to their antibiotic prescription. For some this meant changing to a different mode of delivery, dosage or drug, while for others it meant stopping antibiotics altogether. Again, all participants spoke positively about revisions to their prescriptions, often mentioning that HCPs had taken time to clearly explain and inform them about these decisions:

They upped the dosage frequency, and I think they needed to wait to check because they said we’re giving you a wide-ranging one, but they may need to adapt it…and the dose had changed and it had been explained to me why. (patient 15, female, 50)

Overall, participants reported perceiving the ‘review and revise’ process to be sensible and felt that their experiences matched the description provided by the leaflet. In some cases, participants even felt that the leaflet had helped them to make sense of their experiences.

### Existing knowledge of antibiotics and resistance

All participants had some knowledge of antibiotics and antibiotic resistance and many had past experience of antibiotic treatment. Often this had been a positive experience, both in terms of the prescribing process and the efficacy of treatment, but several participants had previously experienced problems, reporting that certain drugs were less effective or produced side effects. Among those who had more negative experiences, there was still a general feeling of acceptance that they were being prescribed antibiotics because they were the most suitable treatment:

I agree that some antibiotics aren’t great and I know in the past I’ve had some that upset my stomach and had to stop or change them…but I still think you need to take them if you’re that ill and sometimes that outweighs the side effects, and sometimes they can give you something to counteract a side effect. (patient 1, female, 50)

Based both on past experience and references to the media, most participants displayed some knowledge of antibiotic resistance. Although they were not necessarily aware of the mechanisms of how resistance works, there was a general awareness that resistance is a cause for concern and may result in less effective future treatment:

If you use it too much it won’t necessarily work when you do need it, you know? (patient 14, female, 83)

While participants voiced concerns about growing resistance to antibiotic treatments, these appeared to be mitigated by understanding that their current treatment was a necessity. Although they were keen to avoid future resistance and reported that they would be happy to reduce their use of antibiotics if possible, they perceived antibiotics as having been prescribed to combat a serious, often life-threatening, health condition.

### Trust in healthcare professionals

All participants spoke positively about their relationship with HCPs, both in relation to routine care provided by their general practitioner (GP), or their recent care while in hospital. The majority of participants reported being given information about their treatment and condition and being offered the opportunity to ask any questions. Even among participants who had been unaware of the initial prescription, there was a feeling that they had been provided with details about their care as soon as they were in a state to respond to the information. Despite the chance to ask questions, most participants reported that they did not do this as they had either already been given the information they needed, or their condition was improving and they did not have any concerns. Overall, participants appeared to place a large amount of trust in HCPs. There was a sense that HCPs were seen as experts who had patient care as their main priority. This trust in HCPs appeared to mitigate any concerns that participants might have about their treatment, as they were willing to follow expert advice even if it meant changing or stopping an antibiotic prescription:

I put my faith in them, that’s fine. If they stop they stop, I’m quite happy. They said ‘do you mind if we stop them’, so I thought no, you want them stopped, stop them. (patient 5, male, 77)

Several participants explained that although they were happy to be given information about their treatment, they understood that often they did not have a real ‘choice’ about taking antibiotics if they wanted to recover. Overall, there was a pervasive sense among participants that antibiotics had only been prescribed for them because they were really needed.

## Discussion

This study offers novel insights into how patients in secondary care are likely to respond positively to messages advocating a reduction in the use of antibiotics through the ‘review and revise’ approach. Within our participant group, the information leaflet was viewed as both acceptable and useful without causing undue concern. Individuals reported positive experiences regarding antibiotic prescription being changed and stopped. Many participants had prior experience or knowledge of antibiotics and resistance, and generally welcomed efforts to reduce antibiotic usage. There was an overall feeling among participants that HCPs were trusted experts who were providing the most appropriate treatment for their condition.

### Opportunities for improving patient communication and engagement with ‘review and revise’

Our findings suggest that informative and balanced messages are useful in helping patients understand and accept the ‘review and revise’ antibiotic prescribing process. Communicators can ensure that antibiotic messaging is effective in a number of ways. First, messages should incorporate evidence-based information, particularly in relation to antibiotic resistance and the safety and effectiveness of shorter courses of antibiotic treatment [23]. Additionally, they should address common patient misperceptions about the mechanisms of resistance. Previous research has shown that patients appear to view antibiotic resistance as a wider public health threat, rather than a personal one, particularly if they have not taken antibiotics regularly themselves, because they do not see it as something that is transferrable to others [24, 25]. The current study builds on these findings by including a message about how antibiotic resistance can be passed on to family, friends and even pets. Although some patients had questions or concerns about this process and expressed a desire to avoid antibiotic treatment if possible, none reported that they would refuse antibiotic treatment if it had been deemed necessary by an HCP. This suggests that clear and open messages about the spread of resistance may act as welcome and important motivators for the acceptance of the ‘review and revise’ prescribing process among patients.

The long standing and widely held belief that it is important to complete a course of antibiotics to prevent AMR was clearly evident in the current study [24, 26]. This has been challenged by evidence showing that antibiotic treatment courses are often excessive for individual patients [23] and analyses suggesting the belief contributes to overuse of antibiotics and increases selection for AMR [27]. Our study explored reactions to messaging that implicitly suggested that a course of antibiotic treatment may not always need to be completed and found that patients accepted this idea. It may be that these findings are specific to our patient population who had been recently and acutely ill and not always fully aware of all aspects of their treatment. For instance, unlike primary care, a patient in secondary care may be aware that they are receiving antibiotics, but not necessarily the dosage or the length of their initial prescription. While in hospital, patients are closely monitored by HCPs and changes to treatments may be expected during this time. As a result, patients within secondary care may be more open to discussing and accepting changes to their antibiotic treatment. Primary care research in this area has developed strategies to reduce initial prescribing of unnecessary antibiotic courses [28, 29], having shown that antibiotic prescribing increases patient intentions to seek medical care for future illness, compared to either not prescribing, or delayed prescribing [30, 31]. This indicates that antibiotic prescribing decisions can have longer-term effects on health seeking behaviour, although the potential and feasibility of ‘review and revise’ strategies to reduce overuse of antibiotic in secondary care, and how to most effectively communicate this to patients, has not been investigated. Given the positive patient reactions to the concept of ‘review and revise’ within the current study, it may be beneficial to explore how this could potentially facilitate shared clinician-patient decision making.

Our study also highlights the importance of testing messages with the target audience. During the development of our information leaflet, we addressed a number of questions from HCPs and the ethics committee as to the usefulness and responsibility of providing such information to patients. There was some uncertainty about whether patients would actually want an information leaflet and whether it might cause or increase any concerns about antibiotic treatment or resistance. Our findings build on existing research, which has shown that patients within secondary care are keen to receive proactive rather than reactive information about antimicrobials, allowing them to feel more confident and invested in their care [17]. While HCPs may worry about patient reactions, there is a growing body of evidence to suggest that shared decision-making between patient and HCP could have a role to play in educating patients about antimicrobial stewardship and reducing the inappropriate use of antibiotics [32, 25]. There is also an extensive body of literature examining the relationship of trust between patient and HCP and the impact this has on elements such as patient satisfaction and treatment adherence [33, 34]. Our findings are in line with earlier research which shows that secondary care patients place a high level of trust in HCPs and are confident in their ability to prescribe antibiotics accurately and only when necessary [25, 35]. This trust in HCPs combined with the documented want for information and greater patient engagement [17, 35] suggests that patients are open and receptive to messages about the ‘review and revise’ process. Additionally, our findings are consistent with recent research indicating that patients may find it reassuring to be able to share antibiotic treatment information with family [35]. Further research into the timing of messages may also be useful as preferences may vary by clinical population or setting and could alter acceptability. By testing the key components of messaging with target populations, we have the best chance of ensuring maximum effectiveness, while reducing any unintentional, negative impacts [36].

Finally, this study has helped to provide some recommendations for how the leaflet can be best used in the main trial. First, the main trial should make use of the final, updated version of the leaflet, as this was developed based on the patient feedback as detailed in this paper. Second, study sites in the main trial should aim to have a clear plan in place detailing both who will be distributing the leaflet and when it should be provided to the patient. The current study indicated that a lack of time and resources can make it challenging to find a member of staff to distribute the leaflet. As a result, the main study sites may find it useful to address this in their planning to determine the timing and staffing that would be most feasible for their site. Finally, where it is not possible to find the resources or staffing to distribute a leaflet, main trial sites could consider providing the leaflet in another format, such as a poster that is displayed on the wards. Although this may be a less optimal format, it may still help to provide patients access to information that they are keen to receive.

### Strengths and limitations

This in-depth, qualitative study of antibiotic prescribing within secondary care has helped to highlight key themes that should be considered when designing future studies, but it does have some limitations. Recruitment proved challenging due to many participants having been hospitalised for serious health conditions. Although these conditions had improved by the time of discharge and recruitment to the study, often participants were still feeling unwell and in some cases were readmitted to hospital before an interview could take place. As a result, we may have missed a unique set of experiences related to the ‘review and revise’ process among those participants who perhaps went on to receive further antibiotic treatment, which could have altered their perceptions of the process. It would have been preferable to conduct interviews face to face with participants as this could potentially have yielded more in-depth responses; however, this was not practical for this study because of the necessary restrictions around the recruitment process. In addition, due to the unavoidable delay between participant recruitment and interview, not all participants still had a copy of the leaflet by the time of interview. Although every effort was made to ensure that they had the leaflet by sending a replacement copy by post or email, in 1 case this was not possible, and the researcher decided to read the text over the phone rather than potentially lose the study participant. As a result, it is important to consider that this could have had an impact on the responses of that participant; however, they still provided valuable feedback about the leaflet and their overall experiences. It is also important to note that results of the current study are specific to patients within an acute medical unit in a UK secondary care setting and therefore, may not be generalisable to other populations outside the UK or in primary care, where there may be a very different set of clinical issues. While this feasibility study had only one hospital site, the main trial includes 36 sites from healthcare trusts across England, Wales, Scotland and Northern Ireland. Due to the differing characteristics of these varied regions, it is likely that other issues may arise that were not evident within this feasibility study. These may include elements such as the practicalities of who should give the leaflet to patients, when the leaflet should be provided and whether there is sufficient budget to print the leaflet. We would suggest that it would be useful for the main trial to further understand how and if a patient information leaflet advocating the ‘review and revise’ process might be perceived among other hospital populations, e.g. non-acute medical ward. It would also be beneficial to consider how a more diverse patient population across different ages and ethnicities may react to the leaflet as part of the main trial. Finally, it is possible that there may be some response bias among participants who may have felt obliged to provide positive responses regarding their perceptions and experiences.

## Conclusions

Secondary care patients responded positively to clear, factual information about antimicrobials and were keen to receive an information leaflet about antibiotic prescribing and the ‘review and revise’ process. Messages and information about antibiotic treatment coming from HCPs were seen as welcome and trustworthy, as well as being in the best interest of the patient. As such, encouraging HCPs within secondary care to engage patients in greater communication and information provision could provide great advantages in the drive to reduce antibiotic use. Pre-testing messages about antibiotic prescribing and resistance is vital to dispelling any misconceptions either around effectiveness of treatment for patients, or perceptions of how messages may be received. Although it is not feasible to pre-test all messages, for all populations, it remains important to test key components of messaging in order to ensure maximum optimisation and intervention effectiveness.

## Data Availability

The data that support the findings of this study are available on request from the corresponding author (FM). The data are not publicly available due to them containing information that could compromise research participant privacy/consent.

## Abbreviations

HCP: Healthcare professional
AMR: Antimicrobial resistance
NHS: National Health Service
ARK: Antibiotic Review Kit
RCT: Randomised controlled trial
PPI: Public patient involvement
AMU: Acute medical unit
GP: General practitioner
